# A UHPLC–MS/MS Method for Therapeutic Drug Monitoring of Aciclovir and Ganciclovir in Plasma and Dried Plasma Spots

**DOI:** 10.3390/biomedicines9101379

**Published:** 2021-10-02

**Authors:** Federica Pigliasco, Alessia Cafaro, Raffaele Simeoli, Sebastiano Barco, Alberto Magnasco, Maura Faraci, Gino Tripodi, Bianca Maria Goffredo, Giuliana Cangemi

**Affiliations:** 1 Chromatography and Mass Spectrometry Section, Central Laboratory of Analysis, IRCCS Istituto Giannina Gaslini, 16147 Genoa, Italy; federicapigliasco@gaslini.org (F.P.); alessiacafaro@gaslini.org (A.C.); GinoTripodi@gaslini.org (G.T.); giualianacangemi@gaslini.org (G.C.); 2 Department of Pediatric Specialties, Division of Metabolic Biochemistry, Children’s Hospital Bambino Gesù, IRCCS, 00165 Rome, Italy; raffaele.simeoli@opbg.net (R.S.); biancamaria.goffredo@opbg.net (B.M.G.); 3 Pediatric Nephrology, IRCCS Istituto Giannina Gaslini, 16147 Genoa, Italy; albertomagnasco@gaslini.org; 4 Hematopoietic Stem Cell Unit, Department of Pediatric Hematology and Oncology, IRCCS Istituto Giannina Gaslini, 16147 Genoa, Italy; maurafaraci@gaslini.org

**Keywords:** aciclovir, ganciclovir, valganciclovir, valaciclovir, therapeutic drug monitoring, LC-MS/MS, dried plasma spot

## Abstract

The role of therapeutic drug monitoring (TDM) of valaciclovir (VA)/aciclovir (A) and valganciclovir/ganciclovir (VG/G) in critically ill patients is still a matter of debate. More data on the dose–concentration relationship might therefore be useful, especially in pediatrics where clinical practice is not adequately supported by robust PK studies. We developed and validated a new liquid chromatography-tandem mass spectrometry (LC-MS/MS) micro-method to simultaneously quantify A and G from plasma and dried plasma spots (DPS). The method was based on rapid organic extraction from DPS and separation on a reversed-phase C-18 UHPLC column after addition of deuterated internal standards. Accurate analyte quantification using SRM detection was then obtained using a Thermo Fisher Quantiva triple-quadrupole MS coupled to an Ultimate 3000 UHPLC. It was validated following international (EMA) guidelines for bioanalytical method validation and was tested on samples from pediatric patients treated with A, VG, or G for cytomegalovirus infection following solid organ or hematopoietic stem cell transplantation. Concentrations obtained from plasma and DPS were compared using Passing–Bablok and Bland–Altman statistical tests. The assay was linear over wide concentration ranges (0.01–20 mg/L) in both plasma and DPS for A and G, suitable for the expected therapeutic ranges for both Cmin and Cmax, accurate, and reproducible in the absence of matrix effects. The results obtained from plasma and DPS were comparable. Using an LC-MS/MS method allowed us to obtain a very specific, sensitive, and rapid quantification of these antiviral drugs starting from very low volumes (50 μL) of plasma samples and DPS. The stability of analytes for at least 30 days allows for cost-effective shipment and storage at room temperature. Our method is suitable for TDM and could be helpful for improving knowledge on PK/PD targets of antivirals in critically ill pediatric patients.

## 1. Introduction

Optimizing the use of antimicrobial agents is essential for maximizing therapeutic success and limiting the emergence of microbial resistance mechanisms [1]. This is particularly important in critical patients in intensive care units (ICUs) who often manifest an extreme inter- and intraindividual pharmacokinetic (PK) variability [2]. With the growing knowledge on the relationships between antimicrobial drug dosing, pharmacokinetic/pharmacodynamic (PK/PD) exposure, and patient outcomes, there is now a strong rationale to individualize antimicrobial dosing in critically ill patients with the aid of therapeutic drug monitoring (TDM) [3,4,5]. TDM of antimicrobials is encountering an emerging interest, also due to the spread of several multidrug-resistant pathogens. This leads therefore to different approaches even for drugs with a wide therapeutic index. 

Aciclovir and ganciclovir are guanosine nucleoside analogs, 9-[(2-hydroxyethoxy)-methyl]-guanosine and 9-[(1,3-dihydroxy)-2-propoxymethyl]-guanine, respectively.

Aciclovir and ganciclovir and their respective prodrugs, valaciclovir and valganciclovir, are used for the treatment of the herpes simplex virus (HSV) or cytomegalovirus (CMV) infections in critically ill patients [6]. The usefulness of TDM for these antiviral drugs is a matter of debate and is currently under investigation. The availability of reliable analytical methods for the determination of these drugs can help improve knowledge in this field. Here, we report on the validation of a new analytical method based on liquid chromatography coupled to tandem mass spectrometry (LC-MS/MS) for the determination of aciclovir and ganciclovir on plasma and dried plasma spot samples for TDM application. The method presented here was validated following current international guidelines [7] and was successfully applied to clinical samples obtained from pediatric patients under therapy with aciclovir, valganciclovir, or ganciclovir for the prophylaxis or treatment of HSV or CMV infection following solid organ or hematopoietic stem cell transplantation.

## 2. Materials and Methods

### 2.1. Chemicals and Reagents

Aciclovir (ref. A192400) and ganciclovir-d5 (ref. G235002) were purchased from Toronto Research Chemicals (North York, ON, Canada). 

Ganciclovir (ref. Y0001129), Ammonium acetate (ref. 431311), LC–MS/MS-grade methanol (ref. 1.06035.2500) and LC–MS/MS-grade formic acid (ref. 607001000) were purchased from Sigma (Milan, Italy). All reagents had 98% purity. 

All solutions were prepared with HPLC-grade water obtained from a Milli-Q Plus water purification system. HPLC mobile phases were filtered using Millipore membrane filters (0.45 µm) (Millipore, Vimodrone, Italy). 

### 2.2. Calibration Curve, Quality Control, and Stock Solution Preparation 

Aciclovir (A) and ganciclovir (G) were dissolved in water to obtain stock solutions at 2 mg/mL and 1 mg/mL, respectively. A stock solution of ganciclovir-d5 (IS) (1 mg/mL) was prepared by dissolving the substance in HCl (0.1 M). Calibrators and QC were obtained by spiking a pool of blank plasma with analytes from different batches of working solutions of A and G. The 9 point calibration curve, ranging from 0.01 to 20 mg/L, included the LLOQ. QC samples were prepared at the following concentrations: 0.02 mg/L (QC low), 0.5 mg/L (QC medium), and 10 mg/L (QC high).

### 2.3. Human Samples

For method validation purposes, blank samples were obtained from healthy adult volunteers who were not being treated with A or G/VG. 

For A and G quantification in samples, plasma was obtained from both male (*n* = 31) and female (*n* = 19) hospitalized patients with a median age of 9 years (range, 2–21), who were assuming A or G/VG as therapeutic or prophylactic treatment for HSV or CMV infection. Plasma from patients under treatment with A, G, or VG were obtained from leftover samples collected for routine analyses. 

VG is a pro-drug of ganciclovir that opposed to the latter can be exclusively administered intravenously; its route of administration is oral. VG is well absorbed in the gastrointestinal tract and rapidly and extensively metabolized in the intestinal wall and liver to G. Therefore, systemic exposure to VG is transient and low (Valganciclovir, Summary of Product Characteristics). Consequently, in this study, we validated a UHPLC-MS/MS method for exclusive quantification of G in human samples assuming either G or VG. Plasma was separated from peripheral blood collected in tubes with EDTA K3 anticoagulant by centrifuging at 4000× *g* for 5 min. Plasma samples were stored at −20 °C until analyzed. Written consent allowing the collection of leftover samples and the use of clinical and nongenetic data for clinical research was signed by the patients’ guardians. The present paper shows an advancement in the current clinical standard practice using residual material from routine clinical analyses, and it was considered as it was not research but clinical practice. For these reasons, Ethics Committee approval was not required.

### 2.4. Sample Preparation

#### 2.4.1. Extraction from Plasma

A 50 μL aliquot of plasma (calibrators, QCs, and patient samples) was protein precipitated with 150 μL methanol after the addition of 10 µL IS working solution (6 µg/mL). After vortexing, samples were centrifuged at 14,000× *g* for 5 min at 4 °C. The supernatant was then diluted (1:5) with the mobile phase A.

#### 2.4.2. Extraction from Dried Plasma Spots (DPS)

A 50 μL aliquot of calibration standards, QCs, and patient samples were carefully spotted on filter paper using a calibrated pipette and dried at room temperature (25 °C, ±2) for 1 h. Each DPS was punched to obtain a 3.2 mm diameter disk (containing approximately 3.3–3.4 μL of plasma); each disk was placed in a 1.5 mL Eppendorf tube and extracted with 150 μL methanol after the addition of 10 μL IS (6 µg/mL). After 10 min of incubation at 37 ± 1 °C, the samples were centrifuged at 4 °C at 14,000 rpm for 1 min, and then 100 μL supernatant was dried under nitrogen. Samples were reconstituted with 100 µL of HPLC-grade water. Finally, samples were placed in total recovery glass vials, and 5 µL were injected into the UHPLC system.

### 2.5. Chromatographic Conditions

Gradient separation chromatography was carried out on Ultimate 3000 UHPLC Dual-Gradient Pumps (Thermo Fisher Scientific, Milan, Italy) using an Acquity UPLC BEH C18 (2.1 mm × 100 mm, i.d. 1.7 µm, Waters SpA, Milan, Italy), with mobile phase A consisting of 2 mM ammonium acetate and 0.1% formic acid in water and mobile phase B consisting of 2 mM ammonium acetate and 0.1% formic acid in methanol. The gradient started at 5% of phase B, after 0.1 min was programmed to reach 95% in 1.9 min at a flow rate of 350 µL/min; these conditions were maintained for 0.5 min, then the column was washed with 5% B for 2.5 min, for a total run time of 5 min. The column temperature was maintained at 50 °C.

### 2.6. MS/MS Conditions 

Detection was carried out using a TSQ Quantiva Triple Quadrupole system (Thermo Fisher Scientific, Milan, Italy) equipped with an electrospray ionization source (ESI) operating in the positive ion mode (spray voltage at 3500 V). For the optimized MS settings, nitrogen was used as the nebulizer and auxiliary gas, set at 50 and 15 arbitrary units, respectively; vaporizer and capillary temperature setting for both was 350 °C; argon was used as collision gas at a pressure of 1.5 mTorr. 

The specific transition of G, A, and deuterated IS were detected using multiple reaction monitoring (MRM): 256.1→135.1 for G; 226.1→135.1; 110.1; 164.1 for A, respectively. IS was detected using the following transitions: 261.1→110.1; 135.0; 164.1. Although two ion transitions (quantitative and qualitative) are commonly required in quantitative UHPLC-MS/MS, we only chose one ion transition for G because other transitions were too challenged by a poor signal-to-noise ratio. 

### 2.7. Method Validation

#### 2.7.1. Selectivity

Selectivity was investigated by analyzing samples from six healthy volunteers not assuming drugs. Moreover, selectivity was also examined on samples obtained from patients under therapy with A or G. A blank human sample, spiked with both analytes at the LLOQ, and a sample spiked with IS were processed and analyzed using the same method for each batch. Each sample was used to prepare DPS and extracted. The absence of interfering components, in accordance with EMA guidelines, was considered as acceptable when the signal was less than 20% of the LLOQ for G and A and less than 5% for the IS.

#### 2.7.2. Carry-Over

The presence of carry-over was assessed by injecting blank samples in triplicate after the highest calibration standard. As suggested by EMA guidelines, carry-over was considered as acceptable if the signal in the blank sample following the higher standard was less than 20% of the LLOQ and 5% for the IS.

#### 2.7.3. Matrix Effects and Extraction Recoveries

Matrix effect and extraction recovery for G, A, and IS were measured at two different levels (corresponding to the low and high QC) analyzed in triplicate for both plasma and DPS. Matrix effect and extraction recovery were investigated by analyzing 6 lots of blank matrix samples from individual donors. Matrix effects were determined by comparing peak areas of the analytes spiked after extraction to peak areas of pure solution at the same concentration. Extraction recovery was investigated by comparing peak areas of G and A spiked before extraction to peak areas of G and A after extraction.

#### 2.7.4. Linearity

The evaluation of linearity was made by analyzing the calibration curve three times on three non-consecutive days. The peak area ratio of analyte/IS vs. the analyte concentration of each calibration standard were fitted using a 1/*x* weighting factor. We used a weighted (1/*x*) quadratic regression model, because the absolute variation was larger for higher concentrations and the data at the high end of the calibration curve tended to dominate the calculation of the linear regression, often resulting in excessive error at the bottom of the curve [8]. 

**Figure 1:**
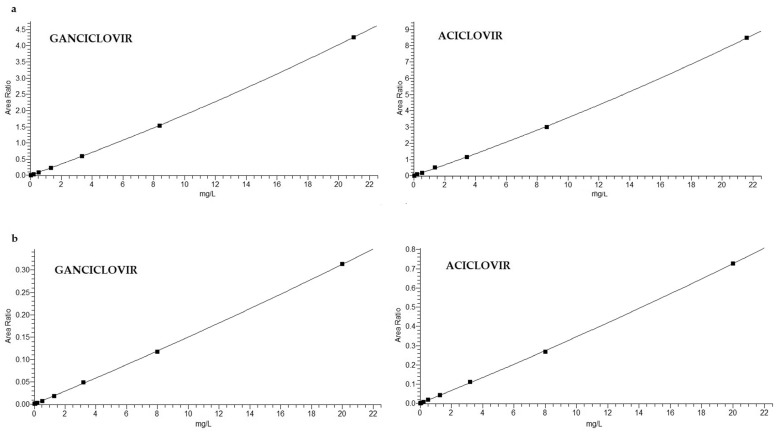
The mean calibration curves (9 point calibration curve) of ganciclovir and aciclovir ranging from 0.01 to 20 mg/L in plasma (panel **a**) and in DPS (panel **b**).

The mean calibration curve statistics were Y = −1.1 × 10^−3^ + 4.4 × 10^−4^ X + 1.4 × 10^−9^ X^2^ with R^2^ = 0.9995 for G and Y = −2.0 × 10^−3^ + 8.9 × 10^−4^ X + 7.6 × 10^−9^ X^2^ with R^2^ = 0.9991 for A in plasma and Y = 1.0 × 10^−3^ + 1.4 × 10^−5^ X + 6.9 × 10^−11^ X^2^ with R^2^ = 0.9995 for G and Y = 1.5 × 10^−3^ + 3.3 × 10^−5^ X + 1.8 × 10^−10^ X^2^ with R^2^ = 0.9997 for A in DPS. The calibration curves were validated in the concentration range 0.013–20 mg/L (Figure 1). The acceptance criteria for the back-calculated concentrations of calibration standards were 15% of the theoretical value, except for the LLOQ (±20%).

#### 2.7.5. Precision, Accuracy, and LLOQ 

Within-run and between-run precision and accuracy were evaluated by testing QC samples five times on three separate days. Accuracy was assessed as the mean relative error (expressed as a percentage) and precision as the coefficient of variation (CV%). The results were considered within the acceptable ranges, within 85–115% and ≤15% of the nominal concentrations for accuracy and precision, respectively. The LLOQ was defined as the lowest concentration that could be measured with a precision ≤20% and accuracy within 80–120% of the nominal concentration. Moreover, the LLOQ should have a signal-to-noise ratio >5. Dilution integrity was determined by diluting 2 and 5-fold (*v*/*v*) the highest calibration standard with blank matrix. Each diluted sample was analyzed fivefold.

#### 2.7.6. Stability

Stability was evaluated by analyzing three replicates of QC low and QC high in DPS and plasma after storage at RT for 4 weeks and at −20 °C for 4 weeks, respectively. As suggested by EMA guidelines [7], stability was considered acceptable if the percentage difference, calculated as the ratio between the concentration measured at each sampling point and the initial concentration, was lower than 15%.

### 2.8. Statistical Analyses

A nonparametric Passing–Bablok regression analysis [9] together with the Pearson correlation coefficient were used to determine the agreement between the concentrations obtained on plasma and those obtained on DPS for both A and G. The 95% confidence intervals (CIs) were calculated for the slope and intercept. The deviation of the response value from its fitted value was evaluated determining the standard deviation of the residuals of the principal component method (RSD). The Cusum test was used to estimate the linear relationship between the two methods.

The Bland–Altman test was then applied to assess the relative differences between the two methods by plotting the percentage differences against the mean A or G values for plasma and DPS [10]. The mean relative differences and the 1.96 standard deviations (SDs) of the differences were calculated. All the statistical analyses were carried out with MedCalc software (MedCalc Software Ltd., Ostend, Belgium).

## 3. Results

### 3.1. Methods Development 

Several screening tests were carried out to optimize the extraction conditions from DPS. Methanol and acetonitrile were tested with or without the addition of a sonication step or thermostatic bath. The extraction procedure, which gave the best results in terms of extraction recovery (ER), is described in Section 2 (Materials and Methods). 

The UHPLC column chosen for the chromatography allowed good separation efficiency and good peak shape [11]. Retention times for G and A were 1.05 min (±0.10) and 1.28 min (±0.10), respectively.

### 3.2. Method Validation

**Figure 2:**
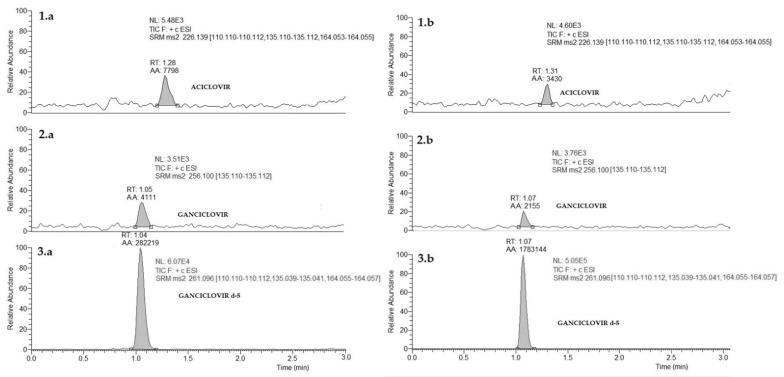
Chromatograms obtained: a calibrator at the LLOQ in plasma (panel **1.a**, aciclovir; panel **2.a**, ganciclovir); deuterated internal standards (panel **3.a**, ganciclovir-d5) and a calibrator at the LLOQ in DPS (panel **1.b**, aciclovir; panel **2.b**, ganciclovir); deuterated internal standards (panel **3.b**, ganciclovir-d5). RT, retention time; AA, automatic area; SN, signal-to-noise ratio; NL, normalized level.

The method was performed according to the EMA guidelines [7]. In particular, no interfering peaks were detected at the specified LC-MS/MS conditions. Carry-over was negligible. The LLOQ resulted in 0.013 mg/L for both A and G. Representative chromatograms obtained are shown in Figure 2. In the entire concentration range, a linear relationship between the analyte’s peak area and the corresponding concentration was achieved (R^2^ = 0.99), and the back-calculated concentration values for A and G were not significantly different from the nominal value (±15%). In particular, CV% was <10% for the eight calibrators and <18% for the LLOQ, complying with EMA guidelines [7].

The results of the intra- and inter-assay precision and accuracy and recoveries were acceptable according to the EMA guidelines [7] (Table 1).

The dilution integrity met the acceptance criterion for accuracy (±15% of the nominal value). Analyses carried out to assess the matrix effect and IS-normalized matrix effect yielded results within acceptable ranges (8–12%). 

Extraction recovery tests results were 90% for A and 98% for G, with a CV% < 15%. Short-term and long-term stability tests (Table 2) demonstrated that both analytes were stable in DPS at RT after 4 weeks.

### 3.3. Clinical Application and Method Comparison 

As suggested by EMA guidelines, all samples were tested in two different analytical runs to evaluate the incurred sample reanalysis precision. 

**Figure 3:**
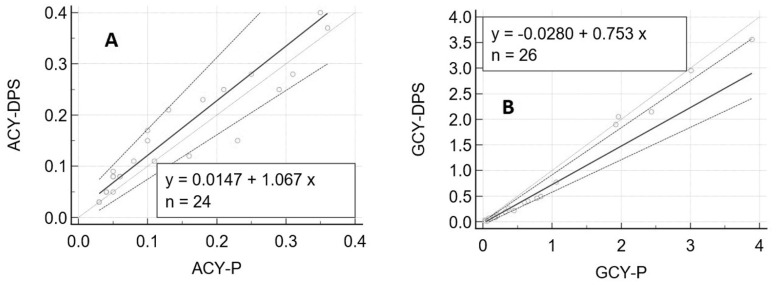
Passing–Bablok correlation plots between aciclovir (**A**) and ganciclovir (**B**) concentrations extracted from plasma. Aciclovir/ganciclovir concentrations (mg/L) measured from plasma are represented on the *x*-axis and aciclovir/ganciclovir concentrations (mg/L) from DPS on the *y*-axis. Thick line, regression line; Thin line, identity line; Dashed line, confidence interval for the regression line.

The results (inter-day RSD = 12%) showed acceptable reproducibility. Figure 3 shows Passing–Bablok correlation plots.

The Pearson’s correlation coefficients were 0.9411 (*p* < 0.0001, 95% CI 0.8668–0.9745) for A and 0.9934 (*p* < 0.0001, 95% CI 0.9852–0.9971) for G.

**Figure 4:**
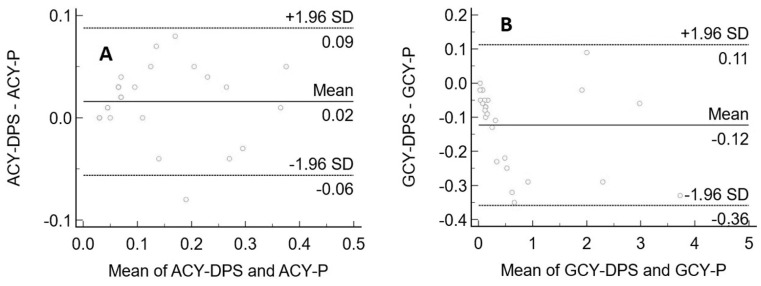
Bland–Altman plots between aciclovir (**A**) and ganciclovir (**B**) concentrations extracted from plasma. On the *x*-axis are the average of the plasma concentration and DPS concentration of aciclovir/ganciclovir (mg/L) measured from plasma and DPS, and the *y*-axis represents the difference between the plasma concentrations and DPS concentrations of aciclovir/ganciclovir (mg/L).

For A, no proportional or additional systemic bias was obtained. For G, the intercept 95% CI included 0 indicating the absence of a constant bias, whereas the slope 95% CI did not include 1, indicating the presence of a slight proportional bias. According to the Cusum test for both A and G, there was no significant deviations from linearity (*p* = 0.22). The results were then evaluated using a Bland and Altman test. The plots obtained are shown in Figure 4. The graphs display a scatter diagram of the differences plotted against the averages of the two measurements. Horizontal lines are drawn at the mean difference and at the limits of agreement, which are defined as the mean difference plus and minus 1.96 times the standard deviation of the differences.

The concentrations of both A and G measured from plasma showed no significant biases with those measured using DPS.

## 4. Discussion

The availability of reliable and robust methods for the determination of drugs and their validation for clinical use can help improving TDM practice, especially when the knowledge regarding the dosing and PK/PD relationship should be expanded. In this paper, we depicted for the first time an LC-MS/MS method for the quantification of aciclovir and ganciclovir in plasma and DPS. This method was validated and applied to routine clinical samples derived from critically ill children who were under antiviral therapy for treatment or prophylaxis of HSV or CMV infection. 

Although there are very few reports in the literature available on this topic, the efficacy of valaciclovir/aciclovir for treatment of HSV infections has been correlated to the level of drug exposure though the evaluation of the area under the curve (AUC) and to free-drug concentration time above the minimum inhibitory concentration (MIC) for the pathogen (%*f*T > MIC) [12,13]. Similarly, for valganciclovir/ganciclovir, a relationship has been demonstrated between drug exposure and efficacy or toxicity, but a general consensus is still lacking. A recent position paper neither recommends nor discourages TDM in critically ill adult patients [1]. In pediatrics, very few reports dealing with this subject are present in the literature [14,15,16].

Several papers showing methods for determination of A or G in human plasma by HPLC or LC-MS/MS have previously been published [11,17,18,19,20,21,22]. Very few of them used a rapid sample preparation protocol based on protein precipitation and validated with human plasma samples for clinical purposes [11,20,22]. Several HPLC methods require time-consuming sample pretreatment, including solid phase extraction (SPE), which are unsuitable for routine use [18,19,23]. 

Thus far, only two publications have evaluated G using dried blood spots (DBS) [17,23], of which only one was validated using clinical samples derived from patients [23]. 

To our knowledge, there are no publications in the literature on the quantification of aciclovir and ganciclovir starting from DPS. 

The use of dried plasma spots could represent a useful tool to facilitate sample storage and shipment to reference laboratories. DPS have already been successfully employed for the quantification of several drugs (anti-HIV drugs, antifungals, antibiotics, antiepileptics, etc.) [24,25,26,27].

DPS is an alternative sampling strategy that consists of collecting plasma samples on filter cards. The disadvantage of this method, if compared to DBS, is that it requires a longer procedure for sample collection due to the centrifugation/decantation step that is necessary to obtain plasma. Conversely, the advantage of this approach is that hematocrit bias is overcome and results coming from this sampling method could be easily used for clinical purposes. We have shown, for the first time, a method for the simultaneous measurement of A and G developed on DPS validated for clinical use. The method’s performance allowed for the rapid and specific quantification of A and G with high accuracy and precision over a wide range of concentrations starting from low (50 μL) volumes of plasma. This aspect is particularly relevant in neonatal and pediatric settings where large volumes of blood and plasma are not always available and/or accessible. Here, thanks to the low volume required for the analysis, we were able to apply this method to samples derived from pediatric patients under therapy with A of G. 

Moreover, another practical advantage of using DPS is based on the opportunity of storing and transporting DPS at room temperature (given the stability of analytes), reducing the risk of the samples’ degradation. Therefore, our method can easily be adopted for TDM application allowing not only for the improvement in our knowledge on the dose–concentration effect of these antiviral agents but also to facilitate the use of these drugs in pediatric patients. This method is reproducible for both plasma and DPS, since the concentrations measured in both matrices were interchangeable thus demonstrating that DPS can be considered as a valid sampling strategy to be adopted to improve TDM of antiviral drugs.

## Figures and Tables

**Table 1:** Results of intra-day and inter-day accuracy and reproducibility assays for plasma and DPS (*n*, replicates; (*n*) = 5). The quality control concentrations were, respectively, 0.01, 0.02, 0.5, and 10 mg/L for LLOQ, QClow, QC medium, and QC high. (SD, Standard deviation; CV%, coefficient of variation percentage).

**Plasma**						
**INTER-DAY**						
	**Ganciclovir**			**Aciclovir**		
	**SD (σ)**	**CV%**	**Accuracy%**	**SD (** **σ)**	**CV%**	**Accuracy%**
LLOQ	0.02	14%	9%	0.02	13%	13%
QC low	0.00	13%	8%	0.00	13%	−14%
QC medium	0.03	14%	−6%	0.03	8%	−12%
QC high	0.42	9%	−6%	0.42	6%	−11%
**INTRA-DAY**						
	**Ganciclovir**			**Aciclovir**		
	**SD (σ)**	**CV%**	**Accuracy%**	**SD (σ)**	**CV%**	**Accuracy%**
LLOQ	0.01	5%	10%	0.01	9%	13%
QC low	0.00	6%	15%	0.00	8%	−14%
QC medium	0.02	7%	2%	0.02	5%	−11%
QC high	0.19	4%	−3%	0.34	4%	−12%
**DPS**						
**INTER-DAY**						
	**Ganciclovir**			**Aciclovir**		
	**SD (σ)**	**CV%**	**Accuracy%**	**SD (σ)**	**CV%**	**Accuracy%**
LLOQ	0.08	14%	12%	0.02	13%	15%
QC low	0.09	13%	14%	001	15%	13%
QC medium	0.01	2%	1%	006	1%	−15%
QC high	0.01	4%	−9%	001	4%	−12%
**INTRA-DAY**						
	**Ganciclovir**			**Aciclovir**		
	**SD (σ)**	**CV%**	**Accuracy%**	**SD (σ)**	**CV%**	**Accuracy%**
LLOQ	0.05	14%	13%	0.04	10%	12%
QC low	0.07	13%	13%	0.08	9%	15%
QC medium	0.05	6%	2%	0.07	6%	−15%
QC high	0.01	2%	−6%	0.01	3%	−15%

**Table 2:** Stability of ganciclovir and aciclovir measured on DPS. Results are expressed as the accuracy and CV percentage (CV%) (CV% is the coefficient of variation percentage).

Ganciclovir		Aciclovir	
T+25 °C			
15 days		15 days	
QC low	10% (7%)	QC low	11% (2%)
QC medium	0.5% (2%)	QC medium	4% (2%)
QC high	2% (2%)	QC high	5% (4%)
**30 days**		**30 days**	
QC low	10% (9%)	QC low	11% (10%)
QC medium	6% (5%)	QC medium	4% (6%)
QC high	5% (4%)	QC high	9% (5%)

## Data Availability

The data presented in this study are available on request from the corresponding author.
